# FORMING AGING RESEARCH TEAMS THROUGH SPECIAL INTEREST GROUPS: THE GRADUATE STUDENT EXPERIENCE

**DOI:** 10.1093/geroni/igad104.1242

**Published:** 2023-12-21

**Authors:** Dana Urbanski, Kamakshi Lakshminarayan, Maritza Steele, Farah Baig, Adrianna Rivera-Leon, Michelle Berning, Joseph Gaugler

**Affiliations:** University of Minnesota, Minneapolis, Minnesota, United States; University of Minnesota, Minneapolis, Minnesota, United States; University of Minnesota, Minneapolis, Minnesota, United States; University of Minnesota, Minneapolis, Minnesota, United States; University of Minnesota, Minneapolis, Minnesota, United States; University of Minnesota, Minneapolis, Minnesota, United States; University of Minnesota, Minneapolis, Minnesota, United States

## Abstract

The study of aging is inherently interdisciplinary, requiring formation of collaborative research teams that span a variety of fields and disciplinary backgrounds. This team approach is essential for answering complex, multifaceted questions about human aging; however, it may serve as a significant barrier to entry for graduate students interested in obtaining training and expertise in aging research. By and large, many graduate students are highly centralized in single academic departments and lack sufficient resources and opportunities to gain interdisciplinary research training and experience. The University of Minnesota Center for Healthy Aging and Innovation (CHAI) has created Special Interest Groups (SIGs) to facilitate team science and interdisciplinary collaboration by providing infrastructure and community for faculty, post-doctoral fellows, and—importantly—graduate students from across campus who have shared interests in aging. Here, we discuss a research project developed and organized by CHAI’s Aging and Chronic Disease Management SIG which has drawn substantial graduate student engagement. Together, eight graduate students representing four University of Minnesota colleges are working with faculty to conceptualize and complete a systematic literature review on caregiving for older adults with hypertension. Drawing on this example, we share early lessons learned as well as key challenges and opportunities for engaging graduate students in interdisciplinary aging research through SIGs. Additionally, we highlight student experiences and perspectives participating in CHAI SIGs, along with recommendations for recruitment and inclusion of graduate students in aging research teams.

